# Salvage living donor liver transplantation after percutaneous transluminal angioplasty for recurrent Budd-Chiari syndrome: a case report

**DOI:** 10.1186/1752-1947-5-124

**Published:** 2011-03-29

**Authors:** Yusaku Shirai, Hitoshi Yoshiji, Saiho Ko, Masaharu Yamazaki, Yasuhide Ikenaka, Ryuichi Noguchi, Chie Morioka, Kosuke Kaji, Yosuke Aihara, Keisuke Nakanishi, Junichi Yamao, Masahisa Toyohara, Akira Mitoro, Masayoshi Sawai, Motoyuki Yoshida, Masao Fujimoto, Masahito Uemura, Yoshiyuki Nakajima, Hiroshi Fukui

**Affiliations:** 1 Third Department of Internal Medicine, Nara Medical University, Nara, Japan; 2 Department of Surgery, Nara Medical University, Nara, Japan

## Abstract

**Introduction:**

Budd-Chiari syndrome is a very rare pathological entity that ultimately leads to liver failure. Several therapeutic modalities, including percutaneous transluminal angioplasty, have been attempted to save the life of patients with Budd-Chiari syndrome. Few reports have described a salvage living donor liver transplantation performed after percutaneous transluminal angioplasty in a patient with acute Budd-Chiari syndrome.

**Case presentation:**

A 26-year-old Japanese man developed severe progressive manifestations, such as massive ascites and hematemesis due to rupture of esophageal varices. After making several investigations, we diagnosed the case as Budd-Chiari syndrome. We first performed percutaneous transluminal angioplasty to dilate a short-segment stenosis of his inferior vena cava. The first percutaneous transluminal angioplasty greatly improved the clinical manifestations. However, after a year, re-stenosis was detected, and a second percutaneous transluminal angioplasty failed to open the severe stricture of his inferior vena cava. Since our patient had manifestations of acute liver failure, we decided to perform salvage living donor liver transplantation from his brother. The transplantation was successfully performed and all clinical manifestations were remarkably alleviated.

**Conclusion:**

In cases of recurrent Budd-Chiari syndrome, the blocked hepatic venous outflow is not always relieved, even with invasive therapies. We have to take into account the possibility of adopting alternative salvage therapies if the first therapeutic modalities fail. When invasive therapy such as percutaneous transluminal angioplasty fails, liver transplantation should be considered as an alternative option.

## Introduction

Budd-Chiari syndrome (BCS) is a rare clinical condition that results from obstruction of the hepatic venous outflow and has a predisposition to thrombosis of the major hepatic veins and/or inferior vena cava (IVC) at points proximal to the right atrium [1]. The manifestations of BCS are variable, such as hepatic enlargement, pain, tenderness, and portal hypertension [2]. When the occlusion is rapid, the patient may present with acute portal hypertensive manifestations, such as progressive massive painful refractory ascites and rupture of esophageal varices. This clinical situation is called acute BCS, and several therapeutic options, including medical treatments (for example, anticoagulants and diuretics) and invasive management, have been employed [2]. Several invasive therapeutic modalities for BCS have been reported, such as percutaneous transluminal angioplasty (PTA), transjugular intrahepatic portosystemic shunt (TIPS), and orthotopic liver transplantation [3]. For acute BCS, these invasive therapeutic options are likely to be the most appropriate modalities [3]. Even with these invasive therapies, however, recovery of the obstructed hepatic venous outflow is not always achieved. In that case, sequential salvage therapeutic modalities should be considered since acute BCS frequently leads to severe life-threatening acute liver failure. We report the case of a patient with recurrent BCS in whom the first PTA was successful, but the second PTA failed to re-open the stenosis of the IVC. Since our patient showed manifestations of acute liver failure, we decided to perform salvage living donor liver transplantation (LDLT) from his brother. The transplantation was successful, and all clinical manifestations were remarkably alleviated.

## Case presentation

**Figure 1:**
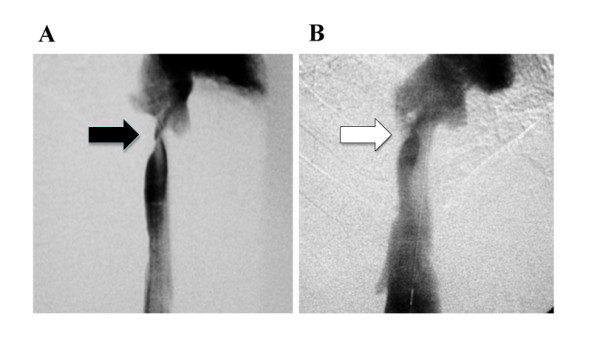
**Angiograms of the IVC before and after PTA**. The IVC showed marked narrowing before PTA (A: black arrow). A balloon catheter was inserted into the right femoral vein, and the stricture of the IVC at the level of the liver was dilated using a 12 × 40 mm balloon catheter. After the first PTA, the stenosis was significantly improved (B: white arrow).

A Japanese 26-year-old man without any relevant medical history had progressive refractory massive ascites of unknown origin. He shortly developed hematemesis. An endoscopy revealed spurting bleeding from esophageal varices, and he was referred to and then admitted into our hospital. He underwent emergency endoscopic injection sclerotherapy (EIS). The laboratory data on admission showed severe liver dysfunction without any suggestion of chronic liver diseases, such as cirrhosis. The hepatitis viral markers, including hepatitis B and C, were all negative. Color Doppler ultrasonography (US) revealed a heterogeneous enlarged liver, shunt vessels between his middle and right hepatic veins (MHV and RHV, respectively), and retrograde flow in MHV. Computed tomography (CT) is a more sensitive modality for showing parenchymal abnormalities. In our case, enhanced CT revealed much clearer heterogeneity of the liver with decreased attenuation in the periphery. It also showed shunt vessels between MHV and RHV. The latter was markedly dilated, and it seemed to have some flow into the IVC, whereas his left hepatic vein was obstructed. Furthermore, sagittal CT images suggested severe stricture of his IVC at the level of the liver. Angiograms confirmed marked narrowing of his IVC at the level of the liver (Figure 1A). Magnetic resonance imaging (MRI) revealed findings similar to those of the CT. From these findings, and the rapid onset of the clinical manifestations, we diagnosed this patient as having acute BCS. Within one week, our patient developed massive hematemesis again. Subsequently, he showed manifestations of acute liver failure, possibly due to "shock liver". Since we could not find a donor for deceased liver donor transplantation (DLDT), we decided to perform PTA first. A balloon catheter was inserted into his right femoral vein, and the stricture of the IVC at the level of his liver was dilated using a 12 × 40 mm balloon catheter. After PTA, the pressure difference between his IVC and his right atrium decreased from 250 mmH_2_O to 50 mmH_2_O, and color Doppler US showed that the blood flow in his MHV had recovered to the normal antegrade flow. His clinical manifestations, such as ascites, dramatically improved, and his liver function also gradually recovered with time. About one month after the PTA, an enhanced 3 D CT examination revealed that the heterogeneity of the liver, the IVC stricture at the level of the liver, and the liver dysfunction had markedly improved. The endoscopic findings of the varices were also alleviated. Our patient was discharged, and regular follow-up was performed.

**Figure 2:**
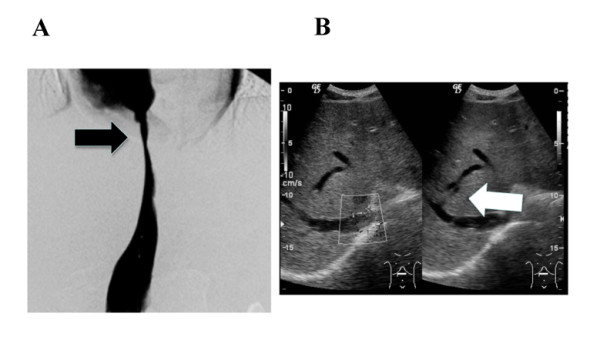
**Images showing various features of re-stenosis after the first PTA**. One year after the first PTA, angiograms of the IVC showed severe stricture of the IVC at the level of the hepatic veins (A: black arrow). Doppler US showed that the RHV and MHV were completely occluded with regurgitation in MHV, and thick shunt vessels from MHV to RHV were noticed (B: white arrow).

**Figure 3:**
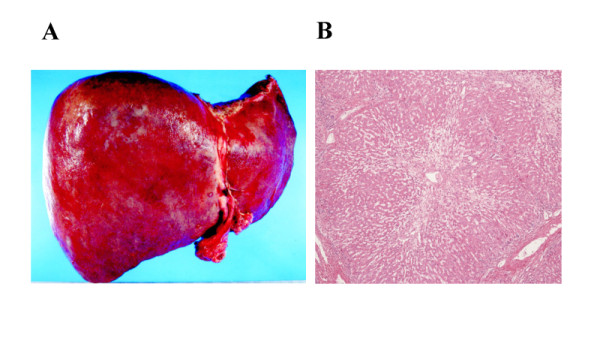
**Photomicrographs of the recipient liver at the time of living donor liver transplantation**. (A) Macroscopically, the recipient liver was swollen and reddish, indicating severe secondary congestion. (B) Microscopically, there was evidence of impaired blood outflow, including congestion, coagulative necrosis, and loss of hepatocytes without the severe inflammatory infiltrates associated with fibrosis development. Original magnification is ×40.

**Figure 4:**
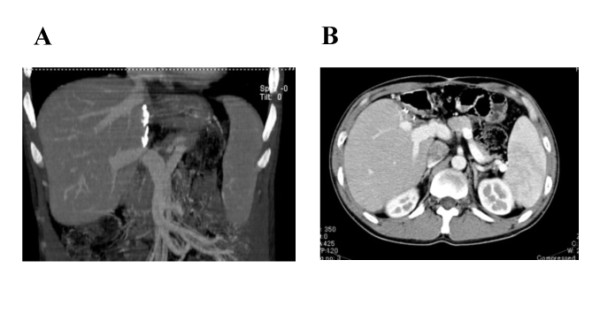
**Multi-slice CT imaging one year after the transplantation**. The sagittal (A) and enhanced images (B) show that the congestion of the liver completely disappeared with normal flow inside the vessels.

After one year, color Doppler US showed that the hepatic venous outflow had deteriorated again, despite our patient being given an anticoagulant after the initial PTA. Sagittal CT images again showed severe stricture of his IVC at the level of the hepatic veins. Our patient then developed massive hematemesis again due to a rupture of the varices. We performed emergency EIS, to control the bleeding. Although we performed PTA again, balloon dilatation was not effective this time. Doppler US showed that his RHV and MHV were completely occluded with regurgitation in his MHV, and thick shunt vessels from his MHV to RHV were noticed (Figure 2). His clinical condition deteriorated over several weeks, including the development of severe hepatic failure. This time, our patient's brother volunteered to become a donor for LDLT. After the physician and surgeon obtained written informed consent from the donor, LDLT was performed using the left lobe of the brother's liver. Macroscopically, the recipient liver was swollen and reddish, indicating severe secondary congestion (Figure 3A). Microscopic examination showed evidence of impaired blood outflow, including congestion, coagulative necrosis, and loss of hepatocytes without severe inflammatory infiltrates associated with fibrosis development (Figure 3B). After the operation, our patient's general condition improved and his manifestations were dramatically alleviated. One year after the LDLT, our patient was discharged with his liver functioning well. The laboratory data indicated a healthy status compared to his status before PTA and surgery (Table 1). Moreover, multi-slice CT imaging showed that the congestion of the liver had completely disappeared with normal flow inside the vessels (Figure 4).

**Table 1:** Laboratory data of the patient

	Before PTA	Before surgery	After surgery
Bilirubin (mg/dL)	1.8	10.6	1.0

Albumin (g/dL)	2.5	2.0	3.3

PT-INR	0.99	1.86	0.74

Creatinine (mg/dL)	0.7	1.3	1.0

MELD Score	9	25	6

Child-Pugh Score	8	14	N/A

## Discussion

BCS is a clinical condition characterized by hepatic venous outflow obstruction due to various underlying causes [1]. BCS is uncommon, with an incidence rate of about one in two and a half million people per year, and current knowledge about its etiology is still insufficient [2]. Most western cases of BCS have a known etiology such as protein-C or -S deficiency, and myeloproliferative diseases, whereas most Japanese cases are idiopathic including acute BCS [4]. The Japanese Ministry of Health and Welfare Research Committee on Aberrant Portal Blood Flow carried out an epidemiological survey and clinical study on BCS, and reported that the majority of BCS patients in Japan are idiopathic, with an obstructing lesion in the IVC [5]. This was also the case in our patient. It has been reported that stenosis of the IVC at the level of the liver is a common cause of non-cirrhotic ascites, which develop within a short period in developing countries [6]. These patients generally have a poor nutritional status, which is now recognized as one of the causes of an immune-compromised state. Patients with such status frequently have thrombophlebitis of the IVC at the level of the liver, which probably results in stenosis or thrombosis of the IVC. Before the onset of symptoms, our patient suffered from alcohol abuse and did not have sufficient nutrition. It may be possible that he had a similar situation of malnutrition, which led to temporary acute stenosis of the IVC.

Clinically, in most cases of BCS, leg edema, abdominal fullness, and subcutaneous venous dilatation over the trunk gradually appear. In some cases, severe clinical manifestations such as progressive refractory ascites and rupture of esophageal varices occur within a short period; a condition classified as acute BCS [7]. Acute BCS often results in fulminant liver failure due to severe liver congestion. In our case, the clinical manifestations developed within a short period accompanied with severe liver dysfunction, which could be classified as acute BCS. Although the general prognosis of acute BCS is poor, the outcome may be fair with accurate early diagnosis and effective treatment. We were able to make an early diagnosis of acute BCS and identify the stricture lesion of the IVC through a combination of several diagnostic modalities, such as Doppler US, 3-D CT, and MRI. After the accurate diagnosis, optimized therapy should be employed for acute BCS. As in our case, about one third of patients harbor short-segment stenosis of either the hepatic veins or IVC. Since such patients are candidates for PTA [2], we first performed PTA and achieved good clinical results for one year. However, it has been reported that PTA has a high level of recurrence and sometimes needs to be repeated. About half the patients who underwent PTA developed re-stenosis within one year. Likewise, our patient had re-stenosis approximately one year after the first PTA[8]. Esophageal varices and ascites developed very quickly along with re-stenosis. We attempted PTA again, but the second time was not effective. The IVC was temporarily dilated but soon re-stenosed. It was reported that an expandable metallic stent (EMS) can be useful for overcoming and reducing the occurrence of re-stenosis or obstructions after PTA [2]. However, in our case, the stenosis level of the IVC and debouch of the hepatic veins were very close. The radiologist in our hospital concluded that we could not employ EMS in this case since sufficient long-term blood flow could not be guaranteed.

Liver transplantation has been used as an alternative to, or after failure of, surgical interventions for BCS [9]. It has been proposed that patients with fulminant hepatic failure consequent to BCS should be listed with the United Network for Organ Sharing system status 1A [10]. However, in Japan, it is extremely difficult to wait for DLDT due to the severe shortage of the number of donors. Although the surgical techniques of LDLT are different from those of DLDT, refined techniques were developed in Japan thanks to the enormous efforts of Japanese surgeons [9]. Although patients with BCS account for only 1-2% of all orthotopic liver transplants, fortunately the department of surgery in our hospital has experience with LDLT. Since our patient's brother suggested becoming a donor for LDLT, we could perform LDLT in the current case. As our patient suffered acute liver failure, all other therapies were less likely to be successful. LDLT was successfully performed, and all the patient's clinical manifestations were significantly alleviated. Therefore, liver transplantation should be considered as one of the alternative options for therapeutic salvage.

## Conclusion

We described the case of a patient with recurrent BCS in whom the first PTA was successful, but the second PTA failed to re-open the stenosis of the IVC. We performed salvage LDLT from his brother. The transplantation was successfully performed, and all clinical manifestations were significantly alleviated. In cases of recurrent BCS, the blocked hepatic venous outflow is not always relieved, even with invasive therapies. We have to consider the possibility of adopting alternative salvage therapies if the first therapeutic modalities fail. When invasive therapeutic modalities such as PTA fail, liver transplantation should be considered as an alternative option.

## Consent

Written informed consent was obtained from the patient for publication of this case report and any accompanying images. A copy of the written consent is available for review by the Editor-in-Chief of this journal.

## Competing interests

The authors declare that they have no competing interests.

## Authors' contributions

YS and HY described the clinical case, obtained informed consent from the patient, conceived the study, participated in its design, assisted in data collection, coordinated and helped draft the manuscript. HY undertook the literature research and contributed to the writing. SK, MY, YI, RN, CM, KK, YA, KN, JY, MT, AM, MS, MY, MF, MU, YN, and HF were responsible for the diagnosis, patient management and review. All authors read and approved the final manuscript.
